# Occupational performance of individuals with Multiple Sclerosis based on disability level in Iran

**Published:** 2019-01-05

**Authors:** Leila Dehghan, Fardin Faraji, Hamid Dalvand, Alireza Shamsoddini, Mohammad Reza Hadian-Rasanani

**Affiliations:** 1 Department of Occupational Therapy, School of Rehabilitation, Arak University of Medical Sciences, Arak, Iran; 2 Department of Neurology, School of Medicine, Arak University of Medical Sciences, Arak, Iran; 3 Department of Occupational Therapy, School of Rehabilitation, Tehran University of Medical Sciences, Tehran, Iran; 4 Exercise Physiology Research Center, Baqiyatallah University of Medical Sciences, Tehran, Iran; 5 Department of Physiotherapy, School of Rehabilitation, Tehran University of Medical Sciences, Tehran, Iran; 6 Brain and Spinal Injury Research Center, Institute of Neurosciences, Tehran University of Medical Sciences, Tehran, Iran; 7 Imam Khomeini Hospital, Tehran University of Medical Sciences, Tehran, Iran

**Keywords:** Multiple Sclerosis, Occupational Performance, Satisfaction, Disability

## Abstract

**Background:** Multiple sclerosis (MS) is a common disease across the world as well as in Iran. Individuals with MS usually experience occupational performance problems that result in limitations in their daily life. This study aimed to determine the occupational performance of individuals with MS based on the disability level in Iran.

**Methods:** In this cross-sectional study, 50 individuals with MS (20 to 50 years old) were recruited through a convenience sampling strategy from different clinics in Arak City, Iran, during 2016-2017. The Persian versions of Canadian Occupational Performance Measure (COPM) and Expanded Disability Status Scale (EDSS) were used to assess the status of occupational performance and level of disability. The data were analyzed using chi-square, Spearman's rank correlation, and Mann-Whitney U tests.

**Results:** The total number of 248 occupations were identified as difficult to perform in the following areas: 125 (50.40%) in self-care, 58 (23.38%) in productivity, and 65 (26.20%) in leisure. In addition, the prioritized occupations (n = 149, median: 3, range: 1-4) had significant difference in the distribution of occupations compared with the non-prioritized occupations (P < 0.0001) and the ratings for performances and satisfactions were generally low. There were significant differences between the occupational performance and level of EDSS.

**Conclusion:** The findings of current study suggest that individuals with MS suffer from widespread problems in the areas of occupational performance, particularly in self-care. The findings emphasize the need for identifying the problems of daily occupations in individuals with MS.

## Introduction

Multiple Sclerosis (MS) is considered as a neurological, progressive, chronic, debilitating, and common disease among young adults.^1^ These individuals may undergo varying degrees of paralysis from lower limb paralysis to upper limb, aphasia, severe visual impairment, motor disease coordination (ataxia), spasm, involuntary eye movement (nystagmus), neurogenic bladder dysfunction, and cognitive disorder.^2^ These impairments can extremely affect function of individuals with MS and lead to limitation of activities and restriction of participations.^3^ Activities and participations are one of the components of International Classification of Functioning, Disability and Health (ICF),^4^ often used identically with the occupations term including performance in three main areas of self-care, productivity, and leisure.^5^ During the last decade, various studies have been performed on the impact of MS on occupational performances. Several studies have shown that individuals with MS often reported problems regarding self-care, productivity, and leisure and they also became mostly unemployed or retired in consequence of this disease,^6^^-^^9^ in addition to ranking low performance and satisfaction in the three areas.^3^

In Iran, the prevalence of MS is estimated to be 11.4%.^10^ Izadi, et al. suggest that Iran has a moderate to high MS prevalence rate, with a recent sharp increase in this rate.^11^ Goal setting of the occupational therapy clinics of individuals with MS focuses on enabling them to continue their engagement in meaningful and purposeful daily occupations depending on the level of disabilities. Therefore, it is necessary to identify the occupations that individuals with MS perceived as difficult to perform to suggest effective interventions.

In spite of a worldwide focus on occupational performances of individuals with MS, there are few studies in this regard which is similar in Iran. Hence, this study aimed to investigate which occupations are perceived as difficult to perform by individuals with MS in terms of self-care, productivity, and leisure areas and also determine the relationship between the level of disability and the mean scores of performances and satisfactions in 20 to 50 years old individuals with MS who had referred to MS Society and rehabilitation centers in Arak City, Iran.

## Materials and Methods

A cross-sectional study was designed and 50 individuals with MS (age: 20 to 50 years) voluntarily participated in the study through a convenience sampling strategy from different clinics in Arak during 2016-2017.

Individuals who met the following inclusion criteria were included in the study: suffering from MS, aged from 20 to 50 years old, free from any mental and emotional or physical disability other than MS such as rheumatoid arthritis (RA) and lupus, and not being at the stage of relapsing phase according to the neurologist's opinion. The exclusion criterion was unwillingness to participate in each stage of study. The study was approved by the Ethical Committee of Arak University of Medical Sciences (Approval No.: IR.ARAKMU.REC.1395.329) and all individuals were informed about the study objectives, and written informed consent was obtained. All individuals primarily were examined by neurologist for determining the type of MS and their disability levels.

***Measurements:*** The data were collected by a demographic questionnaire including age, gender, type of MS, duration of disease, and the duration of neurological rehabilitation. In addition, Expanded Disability Status Scale (EDSS) was completed by a neurologist. This scale measures the severity of disability in individuals with MS^12^ and is suitable to detect the effectiveness of clinical interventions and disease progression.^13^ The disease severity is rated on a scale of 0 (normal) to 10 (death due to MS).^12^ EDSS scores can be specified reflecting the level of disability. These scores reflect between 0 and 5.5 (the early stages of the disease with no assistance during walking), 6.0 and 6.5 (the need for unilateral or bilateral assistance and an ability to walk 100 meters or less with or without rest), 7.0 and 7.5 (severely restricted walking ability), and 8.0 and 8.5 (being unable to walk at all and restricted to bed or chair or perambulated in a wheelchair most of the day).

Similarly, another assessment was used to assign the status of occupational performance in three areas of self-care, productivity, and leisure according to the Persian version of Canadian Occupational Performance Measure (COPM)^14^ by an occupational therapist who was not a member of the research team. 

The COPM is an individualized, client-centered, outcome measure for attaining a client’s self-perception of performance in daily occupations.^15^ The COPM has three occupational areas, each comprising three subcategories: i) self-care (personal care, functional mobility, community management); (ii) productivity (paid/unpaid work, household management, play/school); and (iii) leisure (quiet recreation, active recreation, socialization).

Previous studies with COPM indicated that it was both valid and reliable in using across a variety of disabilities.^16^^-^^18^

In the beginning, the interview process was described to the clients. It is very important that the clients identify activities which they want to do, need to do, or are expected to do in their daily life. The therapist encouraged the clients to think about one day of their life then asked the clients to identify which of the activities were unable to do or were difficult to do now. The importance of each activity was rated on a scale of 1 (not important) to 10 (most important) by clients. In final step, the therapist asked the clients to choose up to five problems that seemed most pressing or important. These identified problems form “prioritized occupations”; then therapist asked the clients to rate each problem on performance and satisfaction on a scale of 1, “not able to do” (performance) or “not satisfied” (satisfaction) to 10, “able to do extremely well” (performance) or “extremely satisfied” (satisfaction).

Adding the performance scores and dividing by the number of identified problems generate a total performance score. Similarly, adding the satisfaction scores and dividing by the number of problems generate a total satisfaction score. These scores will range from l to 10.

In this study, all the steps were carried out individually in a quiet and peaceful environment away from noise. If an individual was illiterate, the therapist or a family member would help her/him. 

The Mann-Whitney U test was used for comparing differences between men and women in the total number of reported occupations in each of the three COPM occupational areas. Spearman correlation test was used to analyze any relationship between level of disability and the mean scores of performance and satisfaction. Chi-square test of independence was used to test whether there was difference between distributions of prioritized occupations and non-prioritized occupations. A P-value of less than 0.0500 was considered to be statistically significant. The data were then analyzed using SPSS statistical software (version 16, SPSS Inc., Chicago, IL, USA).

## Results

The sample consisted of 50 individuals with MS (43 women and 7 men). The mean and standard deviation (SD) of individuals’ age was 38.20 ± 7.40 years. The mean and SD of MS duration was 7.18 ± 3.62 years (the minimum and maximum duration of MS were 1 year and 15 years, respectively). Demographic data of participating individuals with MS are shown in table 1. As illustrated, based on the severity of MS, 33 (66%) individuals suffered from mild disability (EDSS: 0-5.5), 17 (34%) individuals had MS with moderate disability (EDSS: 6-6.5), and none of the individuals with MS had severe disability (EDSS: 7-9.5) (Table 1).

**Table 1:** Demographic characteristics of individuals with multiple sclerosis (MS) (n = 50)

**Clinical features**		**Value**
Sex [n (%)]	Women	43 (86)
	Men	7 (14)
Age (year) (mean ± SD)		38.20 ± 7.40
Type of MS [n (%)]	RRMS	24 (48)
	PPMS	5 (10)
	PRMS	4 (8)
	SPMS	15 (30)
	CIS	2 (4)
Duration of MS (year) (mean ± SD)		7.18 ± 3.62
Severity of MS [n (%)]	Mild disability	33 (66)
	Moderate disability	17 (34)
	Severe disability	0 (0)

MS: Multiple sclerosis; SD: Standard deviation; RRMS: Relapsing remitting MS; PPMS: Primary progressive MS; PRMS: Progressive relapsing MS; SPMS: Secondary progressive MS; CIS: Clinically isolated syndrome

**Table 2:** Description of reported occupations within the three occupational areas of Canadian occupational performance measure (COPM) (n = 50)

**Occupational performance**		**All occupations** * ** (n = 248)**		**Prioritized occupations** ** ** (n = 149)**		
		**n (%)**	**Importance** # **Mean (range)**	**n (%)**	**Performance** ## **Mean (range)**	**Satisfaction** ¥ **Mean (range)**
Self-care	Personal care	41 (16.53)	8.2 (6-10)	28 (18.79)	4.2 (2-6)	3.5 (1-7)
	Functional mobility	41 (16.53)	9.2 (7-10)	22 (14.76)	3.7 (2-9)	3.3 (1-6)
	Community management	43 (17.33)	8.9 (6-10)	32 (21.47)	3.3 (2-7)	2.5 (1-5)
	Total	125 (50.40)		82 (55.03)		
Producti vity	Paid/unpaid work	3 (1.20)	10.0 (10-10)	2 (1.30)	4.0 (3-5)	3.0 (2-4)
	Household management	55 (22.17)	8.8 (4-10)	21 (14.09)	2.4 (2-5)	3.6 (1-9)
	Play/school	0	0	0		
	Total	58 (23.38)		23 (15.43)		
Leisure	Quiet recreation	12 (4.80)	7.5 (5-10)	9 (6.04)	3.7 (2-6)	4.2 (2-7)
	Active recreation	42 (16.93)	5.0 (4-10)	28 (18.79)	2.8 (2-4)	2.2 (2-3)
	Socialization	11 (4.43)	8.1 (7-10)	7 (4.69)	4.4 (2-7)	2.5 (2-4)
	Total	65 (26.20)		44 (29.53)		

* All of the activities that client identifies “unable to do” or “difficult to do now”.

** In final step, up to five problems that seem most pressing or important.

# The client rates the activity in terms of its importance in his or her life. Importance is rated on a ten-point scale.

## The client completes a self-evaluation of his or her current performance in the prioritized occupations using a ten-point scale.

¥ The client completes a self-evaluation of his or her satisfaction with current performance in the prioritized occupations using a ten-point scale.

***Number and rating of reported occupations:*** A total number of 248 occupations were recognized as difficult to perform which 125 occupations (50.40%) pertained to the occupational area of self-care, followed by productivity (58 occupations, 23.38%), and leisure (65 occupations, 26.20%). Most of the problems were found in the subcategory of household management, accounted for 22.17% of all the occupations that individuals with MS perceived as difficult to perform. The rating of importance for them was generally high, between 4 and 10.

The individuals prioritized 149 occupations (median: 3, range: 1-4), within eight of nine COPM subcategories (Table 2). A statistically significant difference was shown in the distribution of occupations among the eight COPM subcategories between the prioritized occupations and the non-prioritized occupations (P < 0.0001).

The mean performance ratings were generally low; the lowest was in household management and the highest in socialization. The range of performance scores in the prioritized occupations were 2-9 and the mean of total performance scores was 5.

Similarly, the mean satisfaction ratings were also generally low; the lowest was in active recreation and highest in quiet recreation. Additionally, the range of satisfaction scores in the prioritized occupations were 1-9 and the mean of total satisfaction scores was 4.

***The reported occupations regarding gender and severity of the disease:*** There was a significant difference between gender and reported occupations for productivity area (P = 0.0010) and women reported significantly more occupations than men; but there was no significant difference for self-care and leisure.

Also, the results showed that there was a significant relationship between EDSS and the mean total scores of performance in the individuals with MS (r = -0.326, P = 0.0210); i.e., as the EDSS level increased, the mean total score of performance decreased. Also, no significant relationship was found between EDSS and the mean total scores of satisfaction in the individuals with MS (r = -0.218, P = 0.1280). However, the negative correlation indicates that if the EDSS level increases the mean score of satisfaction decreases.

## Discussion

In this study, individuals with MS perceived a variety of problems in occupational performance (especially self-care). Furthermore, the most commonly reported occupations were related to household management and the equal numbers of reported occupations were in personal care, functional mobility, community management, and active recreation. This finding is consistent with those of two studies on individuals with MS wherein COPM had also been used and some problems had been reported in these activities.^3^^,^^19^ Esnouf, et al. reported that individuals with MS identified 260 problems that 198 problems were related to self-care.^20^ Similar results have also been reported in other studies of neurodegenerative disorders.^21^^-^^27^ Therefore, it is not surprising that self-care activities are reported as the most problematic activities among the appropriate activities for an independent life; and individuals with disabilities naturally need support to improve their performance in this area.^28^

In our study, approximately 58 activities (23.20%) out of all the reported occupations related to the domain of productivity, wherein household management identified the most commonly occupations. Household management ability which is related to the domain of instrumental activities of daily living (IADLs), is another essential requirement for an independent life. It has been shown that individuals with MS can independently carry out their personal ADLs; however, they are incapable fulfilling the IADLs and have difficulty in living independently.^29^^,^^30^ Therefore, health care professionals should pay attention to the limitations of these individuals in the area of household management. 

In this area, 5.17% of the individuals have reported difficulty in preserving their jobs. It has been well documented that the progression of MS disease affects the ability to work and stay in one's job, even among young people, and may bring about retirement.^8^^,^^9^^,^^30^^,^^31^ These results emphasize the need to address employment issues in individuals with MS.

The number of reported occupations in each of the three subcategories of leisure area accounted for about 29.53% of all the prioritized occupations.

Furthermore, the majority of individuals had identified a high importance to the activities in the leisure area, representing the necessity to pay attention to leisure in individual with MS. Karhula, et al. reported that individuals with MS often experience problems with recreation and leisure activities.^32^ The two studies showed that a majority of the disabled persons perceived one or more severe problems with their participation.^33^^,^^34^ The results point out the importance of accessing to social support along with other factors for enhancing individuals' participation in rehabilitation.

In our study, the low ratings of individuals’ perception of their performance and of their satisfaction suggest that they perceived problems and unsatisfactory in occupational performance. Lexell, et al. indicated similar results using COPM.^3^

A difference with regard to sex was noted in the domain of productivity and the women identified significantly more occupations than the men. As we know, women take responsibility for the wider range of roles such as household management throughout the life course. Therefore, where a woman assumes primary care roles such as household management, more intensive therapeutic support options may be needed.^35^

Also, in the present study, a correlation was found between performance and satisfaction scores with EDSS scores. Similarly, the result of a cross-sectional study suggested that the Functional Independence Measure (FIM) motor score of ADLs in the individuals with moderate to severe MS was associated with the EDSS score.^30^

## Conclusion

In this research, individuals with MS encountered a variety of problems in the domains of self-care, productivity, and leisure. Furthermore, level of their performance and satisfaction was affected by severity of disability (based on the EDSS score). Therefore, it was advocated to evaluate accurately performance and satisfaction in the areas of occupational performance in order to implement suitable intervention approaches for these individuals.

## Notes:

**How to cite this article:** Dehghan L, Faraji F, Dalvand H, Shamsoddini A, Hadian-Rasanani MR. Occupational performance of individuals with Multiple Sclerosis based on disability level in Iran. Iran J Neurol 2019; 18(1): 1-6.
